# Soil Redox Controls CO_2_, CH_4_ and N_2_O Efflux from White-Rot Fungi in Temperate Forest Ecosystems

**DOI:** 10.3390/jof7080621

**Published:** 2021-07-30

**Authors:** Carolina Merino, Ignacio Jofré, Francisco Matus

**Affiliations:** 1 Center of Plant, Soil Interaction and Natural Resources Biotechnology Scientific and Technological Bioresource Nucleus (BIOREN), Universidad de La Frontera, Avenida Francisco Salazar, P.O. Box 54-D, Temuco 01145, Chile; 2 Laboratory of Conservation and Dynamics of Volcanic Soils, Department of Chemical Sciences and Natural Resources, Universidad de La Frontera, Avenida Francisco Salazar, P.O. Box 54-D, Temuco 01145, Chile; ignacio.jofre@ufrontera.cl (I.J.); francisco.matus@ufrontera.cl (F.M.); 3 Network for Extreme Environmental Research (NEXER), Universidad de La Frontera, Avenida Francisco Salazar, P.O. Box 54-D, Temuco 01145, Chile

**Keywords:** N_2_O emissions, redox, temperate forest soil, fungi

## Abstract

Microaerophilic white-rot fungi (WRF) are impacted by oxygen depletion because of fluctuating redox occurrence in southern temperate forest soils of Chile (1500–5000 mm year^−1^). How these conditions influence WRF survival has been scarcely examined. We explored the contributions of WRF to greenhouse gas (GHG) emissions of N_2_O and CH_4_ and soil organic C oxidation (CO_2_) in five sterilized and inoculated forest soils derived from various parent materials and climates. The soil was incubated for 20 days following (i) oxic, (ii) anoxic, and (iii) fluctuating redox conditions. Fungi contributed to 45% of the total GHG under redox fluctuating conditions, including the contribution of bacteria, while the opposite (26%) was valid for oxic treatment. On average, the highest gas emission (62%) was N_2_O for WRF under redox treatment, followed by anoxic (22%) and oxic (16%) treatments, while CO_2_ and CH_4_ emissions followed oxic > redox > anoxic. These data suggest that indigenous microbial WRF communities are well adapted to fluctuating redox milieu with a significant release of GHG emissions in humid temperate forests of the southern cone.

## 1. Introduction

Nitrous oxide (N_2_O) is a powerful GHG~300 times more powerful than carbon dioxide (CO_2_) and 23 times than methane (CH_4_) [1,2]. Under oxic conditions, nitrification dominates over denitrification in mediating N_2_O production [3]. Most nitrifiers are chemoautotrophic organisms (bacteria), abundant in organic matter-rich forest soils [4,5]. Under anoxic conditions, denitrification is the most important for N_2_O production [6]. However, autotrophic nitrifiers (fungi) can also reduce NO_2_^−^ to N_2_O or N_2_, following the oxidation of NH_3_ to NO_2_^−^ under sub-oxic conditions [7]. Lenhart et al. [8] demonstrated that saprophytic fungi continuously released CO_2_ and CH_4_. Although fungi are aerobic organisms in natural environments, they have been adapting their metabolic processes to reduced oxygen (O_2_) levels [9]. For example, white-rot fungi (WRF) growing at tree trunks are more tolerant to low O_2_ and high CO_2_ concentration than other fungi [10,11], because they produce reactive oxygen species (ROS) and extracellular redox enzymes to decompose lignocellulose under restricted O_2_ conditions [12]. Merino, et al. [13] demonstrated that abiotic Fenton reactions coupled to manganese and lignin peroxidase enzymes from WRF interact through synergistic mechanisms to potentiate the oxidation of soil organic matter (SOM) in soils when the O_2_ is depleted. Unfortunately, the authors did not test the SOM oxidation either the GHG emissions contribution under O_2_ fluctuation directly from white-rot fungi, one of the most significant tree wood decomposers in the southern temperate forests [14]. The long-standing hypothesis that N_2_O and CH_4_ generating processes are mediated only by bacteria needs to be revisited since the importance of fungi on N_2_O, and CH_4_ emissions have been scarcely studied.

Our study focused mainly on fungal denitrification and its relationship with other greenhouse gases (CH_4_ and CO_2_). Denitrification has been increasingly investigated, but its community ecology is poorly understood due to the lack of culture-independent tools. Several fungal isolates have been shown N_2_O releases in pure cultures (e.g., [15,16]) and less in soil experiments using selective antibiotics to inhibit fungi or bacteria growth [17,18,19]. These studies found a substantial or dominant role for fungal N_2_O production [20,21,22]. However, the differentiation between the two microbial N_2_O sources (bacteria and fungi) contribution to the N cycle in soil environments needs to be estimated [23]. The most characteristic feature of the fungal-denitrifying system is the involvement of two enzymes; copper-containing nitrite reductase (encoded by the nirK gene) and cytochrome P450 (P450) as nitric oxide reductase (P450nor) in a stepwise manner (NO_2_^−^ > NO^−^ > N_2_O) [24,25,26]. This pathway is widely distributed across subdivisions of fungi (*ascomycota* and *basidiomycota*) but very diverse in terms of activity [27,28]. Nonetheless, N_2_O yields by soil fungi under anaerobic conditions in which nitrate is sequentially reduced responding to organic C oxidation has been recently reported [29,30]. Earlier, Shoun and Tanimoto [31] reported that fungal N_2_O production was generally greater under microoxic (under O_2_ limitation) than under anoxic conditions because both oxygen respiration and denitrification can co-occur [32,33]. However, respiratory NO_3_^−^ reduction arose even after O_2_ was restricted in *Fusarium* spp. strains [22]. In addition, some fungi reported cannot denitrify in the absence of O_2_ [23,34]. Furthermore, the sharp contrast in O_2_ requirement by fungal denitrification suggests that soil fluctuating redox can be a critical factor controlling fungi’s relative contributions to soil N_2_O production. However, quantitative information is still lacking regarding the degree to which fungal contribution is different from the bacteria across a wide range of sub-anoxic conditions.

We hypothesize that the activity of white-rot fungi adapted to soils formed from a wide range of parent materials and precipitation regimes in temperate forests in the southern cone remains significant in GHG (N_2_O, CO_2_, and CH_4_) emissions under fluctuating redox conditions. Therefore, the objective of this study was to determine the importance and contribution of WRF obtained from temperate forest soils mediating the production of GHG emissions in a range of precipitation and parent materials.

## 2. Materials and Methods

### 2.1. Study Sites and Sampling

Five temperate forest soil types were selected, with mean annual precipitation ranging between 1500 and >5000 mm per year. The first sampled soil was a loamy Inceptisol [35] derived from intrusive granodiorite rocks from Nahuelbuta National Park [36]. This soil was developed from ancient *Araucaria araucana* and *Nothofagus pumilio* forests. The second soil was a silty loam Andisol, derived from recent volcanic ash from basaltic and andesitic materials or scoria, and lava [37] from Tolhuaca National Park in the Andes mountains under *Araucaria araucana* and *Nothofagus* spp. The third soil was derived from basaltic-andesitic recent volcanic ash from Conguillío National Park in the Andes under *Araucaria araucana* and *Nothofagus* spp. forests [38]. The fourth soil was a loamy clay Ultisol derived from metamorphic mica-schist materials with illite-kaolinite as dominant clays sampled in Alerce Costero National Park in the Coastal range under *Nothofagus* spp. and *Fitzroya cupressoides* [39]. The final soil sample was an Andisol derived from recent volcanic ash and basaltic scoria deposits with a high content of allophane, imogolite, and ferrihydrite (sandy clay loam) [40]. The soils were collected in the Andes from a primary temperate rainforest of *Nothofagus betuloides* in Puyehue National Park. Soil classification, vegetation, and climate characteristics are presented in the section of results).

Four composite soil samples from each site were extracted from the top Ah mineral horizon (0–15 cm) after removing the litter layer (0–5 cm). The samples were cleaned to remove coarse organic debris in the laboratory and separated into three portions: one portion was stored at 4 °C for microbial and enzymatic analyses, and the second portion was air-dried for further chemical and physical analyses. The last one was stored at 4 °C for the microcosm experiments. 

### 2.2. Analytical Procedure

The pH was directly measured in a soil aliquot in a 1:2.5 suspension of soil:water measured with a pH/mV data logger 850059 (Scottsdale, AZ, USA). Soil organic C was determined using TOC-VCSH (Shimadzu, Kyoto, Japan), and total N was determined by Kjeldahl distillation (VELP, Usmate, Italy). Selective dissolution determination with acid ammonium oxalate 0.2 M at pH 3 for Al (Al_o_) and Si (Si_o_) and pyrophosphate extraction for Al (Al_p_) was obtained using a solution of 0.1 M sodium pyrophosphate [41,42]. Al_o_, extracts indicate the amount of amorphous Al and Fe, and pyrophosphate, the organo-mineral (Al and Fe) association with soil organic matter. All metals and Si extractions were determined by atomic absorption spectroscopy (Perkin Elmer 3110, Waltham, MA, USA). Al saturation and soil texture were conducted as indicated by Sadzawka et al. [41].

### 2.3. Soil Sterilization

To remove the microbial population with resistant structures such as endospores and conidia, soils used for the microcosm experiment (see below) were sterilized in an autoclave for 20 min at 121 °C three times over a period of three days. In addition, soils were fumigated with chloroform vapor in a vacuum chamber during 24 h [43]. Autoclaving was used instead of Gamma irradiation, because it does not create significant changes in the SOM [44]. Gamma radiation was avoided because some reports indicate that it causes Fe reduction and oxidation by increasing the bioavailability of Fe (III) (oxyhydr)oxide minerals, which resulted in increased Fe reduction [45]. Chloroform fumigation of soil has been used since causes a small extractable N by lysing living soil microorganism but does not generate damage to soil organic matter if the fumigation is carried out lesser than 5 days (Brookes et al., 1985). As we were also interested in the potential denitrifying enzyme activity (DEA), chloroform was suggested as an adequate method for separating the synthesis of denitrifying enzymes from cellular metabolism under realistic soil conditions since this fumigation induces minor cell lysis and has a minimal impact on enzymes [46].

### 2.4. Culture Conditions and Fungal Identification

White-rot fungi were isolated from wood logs in the same areas where soil sampling was conducted. The WRF was isolated by transferring small fragments of the fungi fruiting bodies or fragments of decayed wood colonized by fungi on acidified glucose malt extract agar plates (15 g/L agar, 3.5 g/L malt extract, 10 g/L glucose, pH 5.5), and incubated at 25 °C. Pure mycelial cultures were obtained under aseptic conditions, and ITS sequencing identified the strains. The DNA of each strain was extracted using E.Z.N.A.^®^ SP Fungal DNA Mini Kit D5524-01 (Omega, Bio-Tek-Cada, Norcross, GA, USA). The ITS1 –5.8S—ITS2 rDNA was amplified using primers ITS1 and ITS4 [47]. PCR was carried out using 0.1 mM dNTPs, 0.1mmol of each primer, 5 U of Taq DNA polymerase, and the supplied reaction buffer (Promega Inc., Seoul, Korea) in a total volume of 20 μL per reaction. PCR products were sequenced in an ABI PRISM 3730 × l DNA Analyzer System at Macrogen (Seoul, Korea). The nucleotide sequences were compared in GenBank database (Horisawa et al., 2013). The main identified fungi were: *Schizophyllum commune* in Nahuebuta soil, *Ganoderma lobatum* in Tolhuaca soil, *Trametes parvispora* in Conguillio soil, *Stereum hirsutum* in Alerce Costero soil and *Galerina patagónica* in Puyehue soil. For further details, see Table S1, Supplementary Materials.

### 2.5. Inoculum

For inoculum preparation, an Erlenmeyer flask (500 mL) containing 100 mL of sterile modified Kirk liquid medium (per liter: 10 g of glucose, 2 g of peptone, 2 g of KH_2_PO_4_, 0.5 g of MgSO_4_, 0.1 g of CaCl_2_, 500 µM MnSO_4_xH_2_O, 2 mg of thiamine, and 10 mL of mineral salts solution, pH 5.5) was autoclaved at 121 °C for 15 min. The flask was then inoculated with five agar Malt-Extract Agar disks (6-mm diameter) of active mycelia from a five-day-old culture on malt-extract agar cultivated in Petri dishes and incubated at 30 °C for 10 days. Then, the fungal broth culture was homogenized in a sterilized blender for 1 min and used as an inoculum (blended fungal mycelia) [48]. A final concentration 3 × 10^8^ UFC mL^−1^ in 100 µL of isolates cultured were added in each microcosm.

### 2.6. Microcosm Experiment and Gas Sampling

To examine soil redox effects on fungal N_2_O effluxes, 120-mL amber jars with a screw cap septum were used. Approximately 20 g (dry basis) of sterilized and non-sterilized soils were moisture with sterilized water up to 80% water holding capacity. They were packed into microcosms to a bulk density of 0.8 Mg m^−3^ (0.6–0.9 Mg m^−3^ range in all soils). Sterilized soils were inoculated with blended fungal mycelia from each forest soil site. Soils without inculcation were regarded as control. Soil microcosms were purged at the beginning of incubation (12 °C) and after that each sampling time (0.5, 4, 8, 12, 16, and 20 days). Soils (four replicates) were subjected: (i) Anoxic; jars were flushed with N_2_ by two minutes, (ii) Oxic flushed with reconstituted air (21% oxygen, 78% nitrogen, and 1% argon), and (iii) fluctuating redox conditions. The later consisted of four days under oxic followed by four days under an anoxic environment. The redox intervals resulted from preliminary study [49]. For gas sampling (N_2_O, CH_4_, and CO_2_), 10 mL were extracted using a plastic syringe, and this was then injected into a gas chromatograph coupled with thermal conductivity and a flame photometric detector (GC-FID) (Thermo Fisher Scientific™, Austin, TX, USA) with a 30 m DB1-MS column, in the selected ion mode). In addition, microcosms jars were harvested, and at each sampling, soils were homogenized and quickly subsampled for enzymatic and microbial community analysis.

### 2.7. Fungal and Bacterial Contributions to Greenhouse Gas Emissions

To distinguish between bacterial and WRF contribution to GHG emissions in the microcosm experiments, we used antibiotic cycloheximide to inhibit the protein synthesis and thus the soil fungal activity [17,50]. Soil experiments using antibiotics to inhibit fungi and bacteria selectively have been debated since the application of antibiotics to select microbial community functions cannot be specific [51,52]. Therefore, caution should be taken in using antibiotics because they may impact non-target organisms at high concentrations. To prevent the non-target effects of cycloheximide on bacteria, a preliminary experiment was conducted to determine the minimum inhibitory concentrations of the antibiotic that affect fungi but not bacteria. We found that the minimum inhibitory concentrations for cycloheximide of 1.0 mg g^−1^ soil caused the total fungal inhibition (>90% of bacterial survival). Thus, the total contribution of bacteria and WRF from the total GHG emissions was estimated from non-sterilized soil added with the antibiotic to inhibit the soil fungal activity and later inoculated with WRF.

### 2.8. Fungal Abundance

The fungal abundance was measured by ergosterol extraction method [53]. Briefly, the fungal ergosterol from the cell-membrane was extracted from 2 g of moist soil with 100 mL ethanol in an oscillating shaker (250 rpm during for 30 min) [54]. The extracts were subjected to reverse-phase HPLC-UV system (Waters 515) with 100% methanol mobile phase and final detection at 282 nm wavelength [55].

### 2.9. Measurement of Potential Denitrification

The acetylene reduction assay was considered to reflect the potential denitrifying enzyme activity (DEA) in the soil. The assay reflects the enzymatic potential of the soil denitrifying fungi to reduce NO_3_^−^ to N oxides or N_2_ without de novo synthesis of denitrifying enzymes during the laboratory incubation [56,57]. Briefly, 5-g of fresh soil was placed in a glass serum bottle with 5 mL sterile distilled water and sealed with sterilized rubber septa and an aluminum crimp cap. The headspace was flushed with high-purity N_2_ gas to achieve an anoxic condition after two minutes. Thereafter, approximately 15% (*v*/*v*) of the N_2_ saturated headspace was replaced with acetylene gas to inhibit the transformation of N_2_O to N_2_ and the samples were shaken in oscillating shaker (200 rpm) for 30 min to evenly distribute the C_2_H_2_ gas diffusion throughout the soil [49]. The bottles were incubated in the dark at 25 °C for 12 h. Finally, the headspace gas sample was taken and analyzed for N_2_O by a gas chromatograph coupled with thermal conductivity and a flame photometric detector (GC-FID) (Thermo Fisher Scientific™, Austin, TX, USA).

### 2.10. Statistical Analysis

Normal data (*n* = 120) distribution and variance homogeneity were tested for each treatment and soil type following a similar methodology [13]. One-way ANOVA was conducted for cumulative gas sampling (N_2_O, CO_2_, and CH_4_). Repeated-measures ANOVA test was used for the contribution of fungal and bacteria abundance, ergosterol stock, and denitrification enzyme activity during 20 days of incubation. The last three measured variables were plotted as an average of six sampling times. Duncan’s multiple range test was used for multiple comparisons means since all ANOVA tests were significant at *p* < 0.05. All analyses were conducted using the software RStudio (1.1.442).

## 3. Results

### 3.1. Soil Properties

The studied soils (37° S to 40° S) are developed from different parent materials under temperate climate (Table 1) with mean annual precipitation ranging from 1491 to 5000 mm per year^−1^. The pH ranged from 3.6 to 5.8 and the acidic pH was found for Nahuelbuta soil. The soil organic carbon (SOC) varied from 5.9% to 11.4% with similar variation for total *N* (0.3–0.6%). Tolhuaca, Conguillio, and Puyehue soils come from basaltic and andesitic parent materials displaying allophanic clay minerals, all classified as Andisols with high Al complexed with oragnic matter (Al_p_ 9–11 g kg^−1^ soil). The Al_o_ or Fe_o_ indicates that these soils contains high amorphous structures [58]. Inorganic N (nitrate and ammonium) were low values often found in these forest soils with high Al saturation accompanied by low pH (Table 1).

### 3.2. Contribution of White-Rot Fungi to Greenhouse Gas Emissions

**Figure 1:**
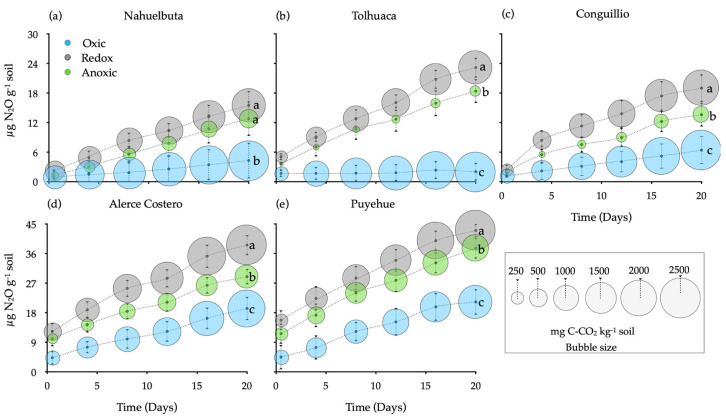
Cumulative N_2_O from temperate forests soils inoculated with white-rot fungi and incubated during 20 days under oxic, anoxic, and fluctuating redox conditions. The bubble size represents the respiratory rate (mg C-CO_2_ kg^−1^ soil). Reference letter in each panel (**a**–**e**) represents the five temperate forest soil sites. Different letters in each panel show significant differences (*n* = 80, *p* < 0.05).

After 20 days of incubation, similar patterns of N_2_O and CO_2_ release were recorded in all soils. The value of the sterilized control soil (without fungi mycelia, ~0.3 µg N_2_O g^−1^ soil) was subtracted from each treatment (Figure 1). However, soil N_2_O fluxes were significantly higher under anoxic or redox fluctuating conditions and did not decrease over the 20 days of incubation (Figure 1). Puyehue soil showed the highest levels of N_2_O, followed by Alerce Costero soil (43.1–38.6 µg N_2_O g^−1^ soil, respectively). The lowest levels of N_2_O were recorded in Tolhuaca soil (2.0 µg N_2_O g^−1^ soil) in oxic conditions, with the opposite being true for the CO_2_.

**Figure 2:**
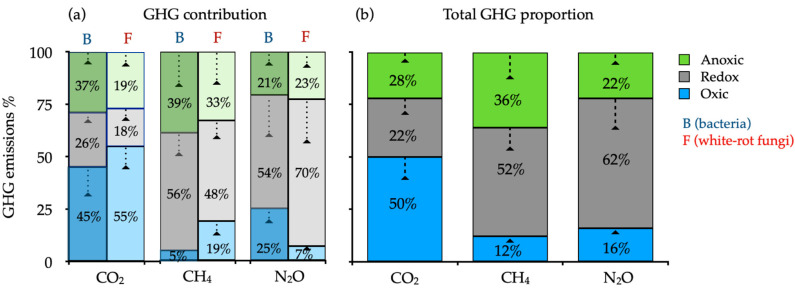
(**a**) Contribution of bacteria and white-rot fungi average to the greenhouse gas emissions (GHG) of sterilized soils and inoculated with white-rot fungi (The letters represent bacteria (B) and white-rot fungi (F) GHG contribution) and (**b**) average of microbial impact (fungi and bacteria) to GHG in temperate rain forests soils incubate for 20 days under oxic, anoxic, and fluctuating redox conditions.

The contribution of white-rot fungi to GHG (N_2_O, CH_4_, and CO_2_) was estimated using an antibiotic (cycloheximide) proxy in which the fungi growth was inhibited to estimate the bacteria emissions. The value of the sterilized control soil (without bacteria and white-rot fungi) was subtracted from each treatment (~8% GHG emissions). The relative contribution of fungi to the N_2_O emissions was 70% compared with only 54% from bacteria under redox 4-day intervals, while in anoxic treatment, fungi and bacteria showed 22% contribution each. In contrast, the CO_2_ and CH_4_ emissions showed the opposite results (Figure 2a). Including WRF and bacteria, approximately 22% of all N_2_O was released via anoxic incubation, and 62% by redox, while under oxic incubation only 16% (Figure 2b). Unlike N_2_O fluxes, the total soil CO_2_ evolutions in soil decreased under redox in 28% and increased under oxic incubation in 50%. Note that in anoxic environment 36% was produced as methane.

**Figure 3:**
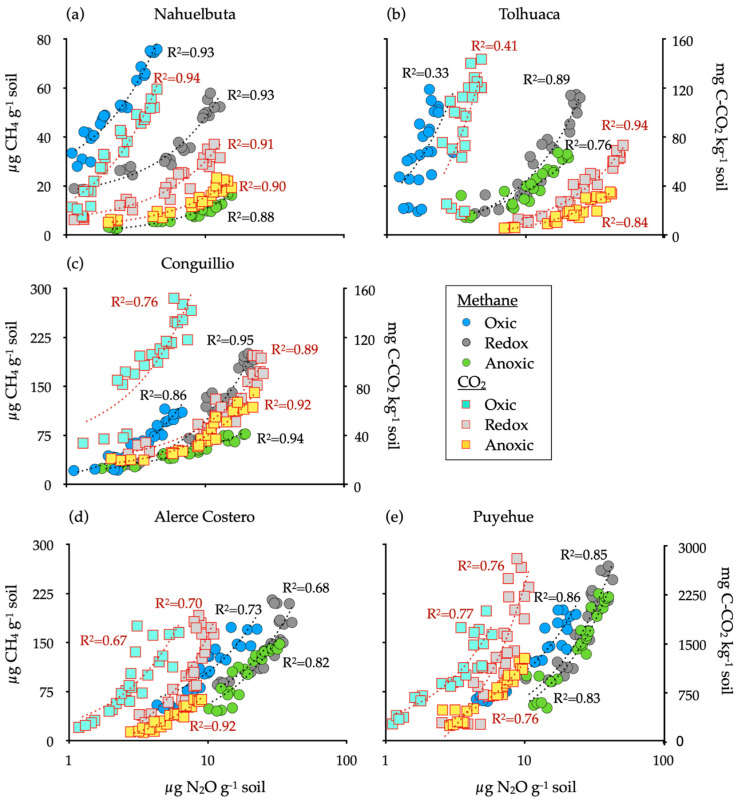
Relationships between N_2_O, CH_4_ and CO_2_ from soils inoculated with white-rot fungi obtained from temperate forests soils incubated for 20 days under oxic, anoxic, and fluctuating redox conditions. Reference letter in each panel (**a**–**e**) represents the five temperate forest soil sites. The relationships were considered significant at *p* < 0.01.

A positive and significant relationship was found between NO_2_ and CH_4_ (*p* < 0.01, R^2^ > 0.68), except for Tolhuaca soil under oxic conditions and between NO_2_ and CO_2_ (*p* < 0.01, R^2^ > 0.67) for all treatments (Figure 3).

### 3.3. Soil Redox and Fungal Abundance

**Figure 4:**
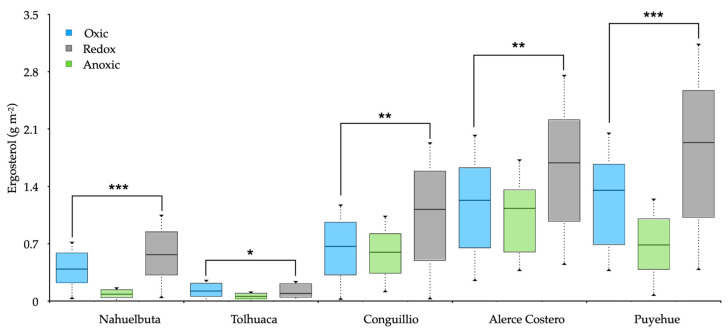
Boxplots for ergosterol stocks in soils inoculated with white-rot fungi obtained from various temperate forests soils incubated for 20 days under oxic, anoxic, and fluctuating redox conditions. The boxplot represents the sample median, and the first and third quartiles. Significant differences at * *p* < 0.5, ** *p* < 0.1 and *** *p* < 0.01.

Ergosterol was detected in variable amounts in all soils but showed significant differences (Figure 4). The value of the sterilized control soil without fungi mycelia (~0.12 g m^−2^) was subtracted from each treatment. Large variations in ergosterol content occurred under O_2_ absence, while fluctuating redox showed increasing values. The ergosterol stocks decreased around 0.4 g m^−2^ between oxic and anoxic conditions and increased from 0.2 to 1.2 g m^−2^ under fluctuating redox conditions (Figure 4).

### 3.4. Soil Denitrification Enzyme Activity

**Figure 5:**
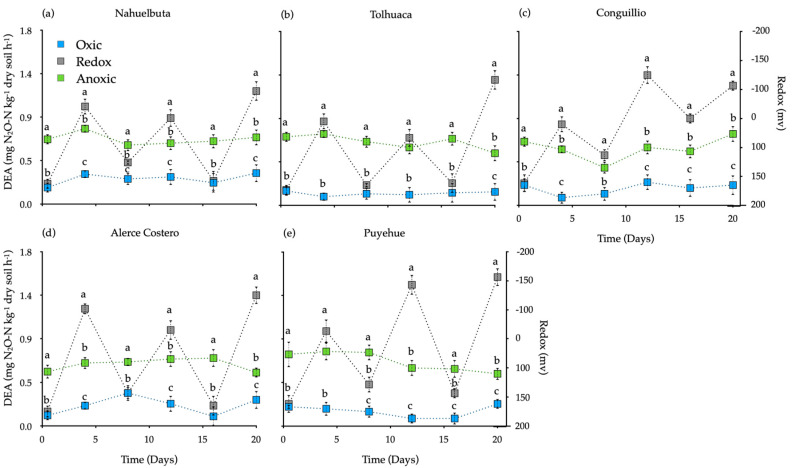
Denitrification enzyme activity (DEA) in soils inoculated with white-rot fungi in various temperate rain forest soils incubated for 20 days under oxic, anoxic, and fluctuating redox conditions. Reference letter in each panel (**a**–**e**) represents the five temperate forest soil sites. Bars indicate standard error of the mean (*n* = 4). Different letters show significant differences (*p* < 0.05).

Denitrifying enzyme activity (DEA, or potential denitrification) rate is shown in Figure 5. As in the other plots, the value of the sterilized control soil without fungi mycelia (~0.3 mg N_2_O-N kg^−1^ dry soil h^−1^) was subtracted from each treatment. Redox conditions had the highest DEA rates (1.17–1.54 mg N_2_O-N kg^−1^ dry soil h^−1^), while oxic incubation showed the lowest. DEA values in the anoxic were higher (>20%) than those in the oxic incubation (*p* <0.05) whose values remained relatively constant through all incubation (Figure 5).

## 4. Discussion

We found that white-rot fungi and bacteria N_2_O production peaked at 62% of the total GHG emissions with variable oxygen concentration, and Fungi contributed 70% of N_2_O emissions compared with 54% of bacteria (Figure 2). In other studies, the microbial emissions of soil N_2_O reached a maximum of 65% of total main emissions under fluctuating redox conditions [59,60].

Bacteria-mediated soil N_2_O emissions that represent the net balance between N_2_O production and consumption [61] contributed at most with 56% for CH_4_ emission during anoxic incubation from the total (white-rot fungi and bacteria) (Figure 2a). As a result, the effects of soil oxygen on bacteria-mediated soil N_2_O emissions are highly dependent on N_2_O consumption. Regardless of differences in vegetation types and parent material of five temperate forest ecosystems, around 70% of N_2_O production in soil was made by the activity of soil fungi under redox conditions. Although this percentage was similar to those reported in tropical forest ecosystems [62,63], the fungal input on soil N_2_O production was comparable to or greater than the bacterial input across the five ecosystems. This new evidence supports that soil fungi are potentially important bio-agents for substantial amounts of soil N_2_O production.

Fungi generally lack N_2_O reductase; therefore, fungal-mediated soil N_2_O emissions are solely dependent on N_2_O production. Small amounts of O_2_ are required for fungal denitrification due to the coexistence of the O_2_ respiration system [32,64]. This is because fungi’s metabolic system can use O_2_ as a substrate in oxic environment and NO_2_— in anoxic conditions [65,66]. Our experiment indicated that fungi preferred fluctuating redox for the maximum N_2_O emission in and this correlated well with both CO_2_ and CH_4_ (Figure 3). Mattila, Mäkinen, and Lundell [12] postulate that white-rot fungi can temporarily tolerate microaerophilic to anoxic growth environments by switching to fermentative metabolism while decomposing wood. In consequence, under limited O_2_ conditions, the viability of cells does not decrease, allowing denitrification under minimal O_2_ requirements [64]. As estimated by ergosterol, the abundance of fungi increased towards south latitude with increased precipitation (Table 1). Ergosterol differences amongst studied sites could be due to some divergence in the composition of the microbial community since different WRF species were found (Table S1, Supplementary Materials). It is also relevant that in the Puyehue soil, the fungi generated a great quantity of N_2_O and DEA, while in the Tolhuaca soil, the N_2_O decreased, and enzymes were produced under redox fluctuating conditions. These differences could be associated with differences in vegetation, average precipitation, including total C and N content, pH, and soil texture [67].

Concerning the soil properties examined in this study, soil pH has the highest difference among the five ecosystems. This can partially explain the differences in the relative contribution of fungi and bacteria to soil N_2_O efflux rate, perhaps due to the impacts of soil pH on the relative abundance and activity of fungi and bacteria. Fungi have been documented to grow over a broader range of soil pH rather than bacteria [68]. Thus, acidic pH (<5.0) may have little influence on fungal biomass [69,70]. However, acidic pH can adversely change the bacterial community [71]. The fungal-to-bacterial biomass ratio has been found to increase with reduced soil pH [70,72,73]. In our study, acidic soil pH in temperate forest soil might shift soil microbial. The differential DEA in the soil forest depended presumably on the changes in abundance of with-rot fungi (ergosterol) as identified (*Schizophyllum commune* in Nahuebuta, *Ganoderma lobatum* in Tolhuaca, *Trametes parvispora* in Conguillio, *Stereum hirsutum* in Alerce Costero, and *Galerina patagónica* in Puyehue soil, see Table S1, Supplementary Materials) and variations in soil SOC, due to the lack of available C as an energy source for denitrifies to express DEA. The fundamental relations of substrate load differences with the microbial density functions have been reported elsewhere (e.g., [74]). According to the ergosterol and DEA (Figure 4 and Figure 5), with-rot fungi yielded more N_2_O emissions in Puyehue and Alerce Costero soils. As mentioned above, fungi accounted for between 22% and 62% of the total emission, including bacteria in redox fluctuating conditions. However, the contribution of N_2_O and CH_4_ from fungi decreased as oxygen increased, suggesting that the microaerophilic requirement of fungal denitrification could not be satisfied with high oxygen. This inverse relationship between the contribution of fungi and O_2_ was in agreement with the findings of other studies [75,76]. In fact, [76] documented that fungi yielded more N_2_O in redox fluctuating conditions, and bacteria produced more N_2_O in anoxic environments. Changes in soil aeration were well controlled in the experiment because the fungi CO_2_ respiration decreased significantly in O_2_ depletion from 65% to 90%. This suggests that fungal heterotrophs were less tolerant than bacteria to more anoxic conditions. This uneven response to O_2_ stress could partially explain why the contribution of fungi to N_2_O was reduced at the highest redox fluctuation.

## 5. Conclusions

This study provides more detailed assessments of fungal contributions to soil N_2_O fluxes and other GHG such as CO_2_ and CH_4_ in a microcosm experiment under redox fluctuating conditions given the limited number of studies, particularly in temperate humid forest soils. On average, white-rot fungi isolated from five forest soils (Andisols, Inceptisol, and Ultisol), most from *Nothofagus* spp. and *Araucaria araucana* forests, greatly contributed to N_2_O emission under anoxic and oxic fluctuation (redox fluctuating conditions) with more than 62% of the total GHG. Fungi accounted for 70% and bacteria 54% of the N_2_O emissions. In comparison, these values were 7% and 25% in oxic incubation. The present results supported the previous one by Merino, Kuzyakov, Godoy, Cornejo, and Matus [13] where isolated ligninolytic enzymes from white-rot fungi were very active lignin decomposers under anoxic incubations in similar forest soils. The present study also supported the fungal dominance on N_2_O production in acidic soil with high organic C content under fluctuating redox conditions.

## Figures and Tables

**Table 1:** Study sites and soil characteristics.

Analysis	Units	Nahuelbuta	Tolhuaca	Conguíllio	Alerce Costero	Puyehue
Coordinates		37°47′ S–72°59′ W	38°12′ S–71°48′ W	38°40′ S–71°39′ W	40°12′ S–73°26′ W	40°47′ S–72°12′ W
Parent materilas		Granitic	Basaltic-Andesitic	Basaltic-Andesitic	Metamorphic, mica-schits	Basaltic-Andesitic-scoria
Soil Order ^1^		Inceptisol	Andisol	Andisol	Ultisol	Andisol
MAT ^2^	°C	13.3	8.6	10.5	9.5	9.2
MAP ^3^	mm a^−1^	1491	3173	2500	4000	5000
Elevation	m a.s.l.	1000	2.806	1400	1048	800
Vegetation ^4^		AA, NP	AA, ND, AP	AA, ND	DW, LP; NN, NP, PN, SC	NB
SOC ^5^	%	10.4 ± 0.02	9.2 ± 0.4	5.9 ± 0.2	9.7 ± 0.2	11.4 ± 0.3
N total	%	0.47 ± 0.01	0.3 ± 0.02	0.37 ± 0.01	0.4 ± 0.00	0.6 ± 0.03
C:N ratio	Unitless	24.3	23	15.9	23.8	19.1
pH wáter	Unitless	3.6 ± 0.2	5.5 ± 0.2	5.8 ± 0.3	4.5 ± 0.2	5.1 ± 0.1
NO_3_^−^	mg kg^−1^	2.0 ± 0.2	2.8 ± 0.3	1.8 ± 0.2	2.6 ± 0.4	3.1 ± 0.1
NH_4_^+^	mg kg^−1^	2.2 ± 0.1	3.3 ± 0.1	2.6 ± 0.4	3.1 ± 0.2	4.2 ± 0.2
Al_p_^6^	g kg^−1^	0.7 ± 0.1	3.3 ± 0.5	1.8 ± 0.9	5.7 ± 0.1	11.2 ± 0.2
Fe_p_^6^	g kg^−1^	7.0 ± 0.2	3.5 ± 0.7	3.1 ± 0.09	9.0± 0.4	7.8 ± 0.3
Al_o_^7^	g kg^−1^	7 ± 0.02	9.0 ± 1.5	7.5 ± 1.5	5.7 ± 0.02	11.0 ± 1.5
Fe_o_^7^	g kg^−1^	6.1 ± 0.2	8.1 ± 0.3	6.8 ± 0.2	2.3 ± 0.3	14.0 ± 0.1
Si_o_^7^	g kg^−1^	2.2 ± 0.4	2.8 ± 0.2	1.2 ± 0.1	1.4 ± 0.1	3.1 ± 0.1
Al saturation	%	80	61.2	94.1	93.5	22.4
Clay type ^8^		K	Allophane	Allophane	Q, I, K	Allophane
Texture ^9^		L	SL	SL	CL	SCL

^1^ Soil Survey Soil Survey Staff [35]; ^2^ Mean annual temperature; ^3^ Mean annual precipitation; ^4^ AA: *Araucaria araucana*; DW: *Drimys winteri* J.R; LP: *Laureliopsis philippiana* (Looser) Schodde (Monimiaceae); NB: *Nothofagus betuloides* (Mirb); NN: *Nothofagus nitida* (Phil); NP: *Nothofagus pumulio*; ND: *Nothofagus dombeyi*; PN: *Podocarpus nubigena* Lindl; SC: *Saxegothaea conspicua* (Lindl.) and WT: *Weinmannia trichosperma* Cav.; ^5^ Soil organic carbon; ^6^ Pyrophosphate extractable Al and Fe Sadzawka et al. [41]; ^7^ Oxalate extractable Al, Fe and Si; ^8^ Q:quartz, K: kaolinite, I: illite Sadzawka et al. [41]; ^9^ SCL: sandy clay loam, CL: clay loam, L: loam, SL: silty loam, Sadzawka et al. [41].

## Supplementary Materials

The following are available online at https://www.mdpi.com/article/10.3390/jof7080621/s1, Table S1: Isolated white-rot fungi strains and ITS rDNA identification.
