# Supplementary material for: Soil Redox Controls CO2, CH4 and N2O Efflux from White-Rot Fungi in Temperate Forest Ecosystems
Source: J Fungi (Basel). 2021 Jul 30;7(8):621. doi: 10.3390/jof7080621 (PMC8398011; doi:10.3390/jof7080621)
Supplement: Supplementary file 1 [file jof-07-00621-s001.zip › jof-1280876-supplementary.pdf]

Journal of Fungi

Supplementary

Soil Redox Controls CO_2, CH_4 and N_2O Efflux from White-Rot Fungi in Temperate Forest Ecosystems

Carolina Merino [1,2,3,*], Ignacio Jofré [2,3] and Francisco Matus [2,3]

[1] Center of Plant, Soil Interaction and Natural Resources Biotechnology Scientific and Technological Bioresource Nucleus (BIOREN), Universidad de La Frontera, Avenida Francisco Salazar, P.O. Box 54-D, 01145 Temuco, Chile
[2] Laboratory of Conservation and Dynamics of Volcanic Soils, Department of Chemical Sciences and Natural Resources, Universidad de La Frontera, Avenida Francisco Salazar, P.O. Box 54-D, 01145 Temuco, Chile; ignacio.jofre@ufrontera.cl (I.J.); francisco.matus@ufrontera.cl (F.M.)
[3] Network for Extreme Environmental Research (NEXER), Universidad de La Frontera, Avenida Francisco Salazar, P.O. Box 54-D, 01145 Temuco, Chile
* Correspondence: carolina.merino@ufrontera.cl

Table S1. Isolated white-rot fungi strains and ITS rDNA identification.

Sample origin	Closest related species	Similarity (%)	Accession Number
Nahuelbuta	*Schizophyllum commune*	100	AF249358.1
Tolhuaca	*Ganoderma lobatum*	100	AH008103.2
Conguillio	*Trametes parvispora*	99.54	MK736989.1
Alerce Costero	*Stereum hirsutum*	100	AF506479.1
Puyehue	*Galerina patagonica*	99.61	KM975403.1
